# Imaging of gallbladder metastasis

**DOI:** 10.1186/s13244-021-01049-8

**Published:** 2021-07-14

**Authors:** Giulio Cocco, Andrea Delli Pizzi, Raffaella Basilico, Stefano Fabiani, Alessio Lino Taraschi, Luca Pascucci, Andrea Boccatonda, Orlando Catalano, Cosima Schiavone

**Affiliations:** 1 Unit of Ultrasound in Internal Medicine, Department of Medicine and Science of Aging, “G.D’Annunzio” University, Chiety, Italy; 2 Department of Neurosciences, Imaging and Clinical Studies, “G.D’Annunzio” University, Chiety, Italy; 3 Istituto Diagnostico Varelli, Naples, Italy

**Keywords:** Gallbladder, Neoplasm metastasis, Ultrasound imaging, Tomography (X-ray computed), Magnetic resonance imaging

## Abstract

Gallbladder metastasis (GM) is a rare condition, often with a late diagnosis or detected upon autopsy. There is no extensive literature on the imaging diagnosis of GM. Here we present a comprehensive review of the literature with the aim of helping to interpret the clinical findings and imaging features of such patients. Few studies on GM are reported in literature. GM by melanoma accounts for about 55.6% of cases. The remaining cases origin from breast cancer (13.6%), hepatocellular carcinoma (13.6%), renal cell carcinoma (6.8%), lung cancer (4.5%), lymphoma (3.5%) and gastric cancer (2.4%). The most common clinical presentation of GM is abdominal pain from cholecystitis due to obstruction of the cystic duct. The main ultrasound (US), computed tomography (CT) and magnetic resonance imaging (MRI) findings that clinicians and radiologists should consider in their everyday medical activity were discussed. The diagnosis of GM was often achieved through a combination of more than one imaging modality. In more than 90% of cases, the diagnosis of GM is often late and combined with other organs involvement in the terminal stage of the malignancy. The knowledge of the clinical features and different imaging techniques through careful evaluation of the gallbladder can help to achieve early diagnosis and avoid misdiagnosis or false negative results.

## Keypoints

- GM can occur in melanoma, renal, breast, hepatocellular, lung and gastric cancer.
- GM diagnosis is often achieved through a combination of more imaging modalities.
- Knowledge of different imaging features can help to achieve an early diagnosis.

## Background

Gallbladder metastasis (GM) is a rare condition, often with a late diagnosis or detected upon autopsy [1, 2]. Few studies on GM are reported in literature. The involvement of the gallbladder in these cases is usually described in the context of diffuse metastatic disease with a very poor prognosis, and it is not commonly identified in live patients [3, 4]. Moreover, GM by melanoma accounts for about 55.6% of cases, the remaining cases origin from breast cancer (13.6%), hepatocellular carcinoma (13.6%), renal cell carcinoma (6.8%), lung cancer (4.5%), lymphoma (3.5%), gastric cancer (2.4%) [3–10]. The most common clinical presentation of GM is abdominal pain from cholecystitis due to obstruction of the cystic duct. The diagnosis of GM was often achieved through a combination of more than one imaging modality. In more than 90% of cases, the diagnosis of GM is often late and combined with other organs involvement in the terminal stage of the malignancy [3–76]. With this comprehensive review we present an overview on GM pointing out the main Ultrasound (US), computed tomography (CT) and magnetic resonance imaging (MRI) findings that clinicians and radiologists should consider in their everyday medical activity.

## Melanoma

Melanoma is an aggressive cancer that usually arises from the skin; it is highly metastatic and originates from melanocytes—dendritic pigment-containing cells located in the basal layer of the skin, eye, mucosa of the upper respiratory tract, gastrointestinal tract and lymph nodes [5]. Only 2–4% of patients affected by cutaneous melanoma have gastrointestinal metastases; the most common sites are the small bowel, colon and stomach [3]. Melanoma metastatic to the gallbladder, although rarely encountered, accounts for about 55.6% of cases of tumor metastases found in this organ [3–46]. Its propensity for hematogenous spread to abdominal locations could explain its occasional seeding of the gallbladder. Clinically, these tumors are often asymptomatic, as evidenced by the discrepancy between the number of published case reports and the rate of detection at autopsy [5–7]. As would be expected, the most common presentation for GM from melanoma is cholecystitis [7–11], most likely due to obstruction of the cystic duct by the tumor mass [12]. Dong et al. [13] reported that 21.1% of gallbladder melanoma cases (4 out of 19) had primary lesions and 27.3% (3 out of 11) had symptomatic metastatic disease [6, 8–12, 15–35]. Cases of associated jaundice and biliary fistulae have also been reported [15, 30]. Several modalities have been employed to assess tumors of the gallbladder, including US, CT, MRI [12, 14, 19, 31, 36–41]. CT currently represents the first choice and the most widely used method for the staging, surveillance and assessment of therapeutic response in melanoma patients. However, US may represent the initial examination for the assessment of the gallbladder in patients with an unknown melanoma. Moreover, contrast-enhanced US (CEUS) and MRI can be useful when CT findings are inconclusive [77, 78].

CT and MRI may show focal thickening of the gallbladder wall or intraluminal masses with arterial enhancement after endovenous contrast administration similarly to other hypervascular metastasis. GM from melanoma are generally larger than 1 cm and attached to the gallbladder wall. If the mass involves the biliary tree, ductal dilation and intraluminal masses may be visualized. The melanin content results hyperdense on unenhanced CT images and hyperintense on T1-weighted (T1w) MRI thus supporting the differential diagnosis with other primary or secondary gallbladder lesions. However, in case of low melanin content or in presence of hemorrhage and necrosis the signal may be variable [79]. Moreover, the diffusion weighted imaging (DWI) shows restricted diffusion. On US, the typical appearance of GM due to melanoma is that of single or multiple broad base mass with a low-moderate echogenicity due to the low reflectivity of melanin. Moreover, color doppler and CEUS may play a complementary role for the diagnosis of malignancy [79]. In fact, although these findings are non-specific for melanoma, the presence of flow signal on Color Doppler rules out the presence of biliary sludge and cholesterol polyps while an early wash-out on CEUS suggests malignancy [80].”

## Renal cell carcinoma

Renal cell carcinoma (RCC), about 70% of which is caused by clear cell RCC, has a propensity to metastasize to uncommon sites, even many years after diagnosis of the primary lesion. About one-third of patients with this cancer develop metastases [47]. However, even in these patients, GM is extremely uncommon [47]. Very few cases of RCC metastatic to the gallbladder are reported in literature [47–52]. In these studies, diagnosis was made more often using a combination of more imaging methods including US, CT, MRI, and endoscopic US. Among the cases reported in the literature, GM had no gastrointestinal symptoms in five cases [48–52]. Gastrointestinal symptoms, such as abdominal pain, nausea and vomiting, were reported in only one case [51]. Two peculiar clinicopathological features are recognized in gallbladder metastases from renal cell carcinoma. First, differently to primary gallbladder tumors, there is a clear male predominance and the association with gallstones is low in GM from renal cell carcinoma. Second, the hematogeneous spread to the gallbladder usually develop as serosal implants and grow progressively as intraluminal pedunculated masses [47]. Imaging are usually non-specific. Compared to primary tumors, GB metastasis from renal cell carcinoma shows a hypervascular pattern with early wash-in and wash-out [81]. US represents a useful first-line method, highlighting non-specific findings such as masses at the body of the gallbladder with a smooth surface and slightly inhomogeneous inner echoes. On MRI, GB metastasis show a high signal intensity on T2-weighted images (T2w) and a restricted diffusion on DWI with lower apparent diffusion coefficient (ADC) than benign lesions. T1w MR findings also correlated with the histologic appearance. A rim of high intensity on T1w images corresponded to marked subepithelial hemorrhage; hypervascularity and intratumoral hemorrhage are well known characteristic findings of renal cell carcinoma [47, 51, 52].

## Lung cancer

Most gallbladder metastases by lung cancer are often detected metachronously. GM from non-small cell lung cancer (NSCLC) can be frequently symptomatic presenting as acute cholecystitis [53]. This finding may be due to the aggressiveness of the primary tumor. Imaging findings are non-specific. Due to the hematogenous diffusion to the gallbladder, GM initially occur as small flat nodules below the mucosal layer and then grow as pedunculated nodules [47]. Usually GM are large hyperechoic (US) or hyperdense broad-based lesions (CT), greater than 1 cm in diameter and rarely associated with gallstones [54–56].

## Breast cancer

Most of the rare case of GM from breast cancer are related with lobular histology [59, 82]. According to literature data, there is no evidence of a particularly useful imaging method with typical features for breast carcinoma metastases. Conventional diagnostic methods are non-specific. In fact, US, CT and MRI may show an enlarged gallbladder with or without endoluminal sludge, signs of wall inflammation and inhomogeneous wall masses. Among the cases reported in literature, all cases of GM were associated with symptoms of cholecystitis. Medical history, location of pain, fever, leukocytosis, and presence of jaundice are necessary to pursue the final diagnosis [64–67].

## Hepatocellular carcinoma

Hepatocellular carcinoma (HCC) can metastasize to the gallbladder by four possible routes: the hematogenous route via the portal venous system, usually with portal vein tumor thrombosis (1), the lymphatic route (2), direct invasion (3) and peritoneal dissemination (4) [83]. Imaging findings are non-specific and a preoperative diagnosis of GM from HCC is difficult. About half of the cases may develop acute cholecystitis. GM can appear as an asymmetric gallbladder wall thickening on US, CT and MRI. Moreover, tumor thrombosis in the portal vein or in the cholecystic veins can be observed, with or without an apparent tumor mass in the gallbladder wall [69–72].

## Gastric cancer

Only three studies on GM from gastric cancer were found in literature. In 2009 Yoon et al. analyzed 417 cases of gallbladder malignancies and firstly reported the gastric origin of GM. More in detail, eight cases of GM from gastric cancer (seven adenocarcinoma and one signet ring cell carcinoma) were reported over a total of 20 GM. The other two studies were case reports. One of them described a case of GM due to gastric adenocarcinoma and the other one reported on a GM due to signet ring cell gastric carcinoma presenting with an acute cholecystitis. In fact, patients can be asymptomatic or present obstructive jaundice, when the tumor is located in the common bile duct, and right upper abdominal pain due to acute cholecystitis. Imaging findings were non-specific and included US or CT asymmetrical wall thickening and fixed filling defects in the gallbladder lumen [74, 75, 84].

## Lymphoma

GM due to lymphoma include B-cell lymphoma, mantel cell lymphoma and T-cell lymphoma. In general, they have a better prognosis than the other GM. The histology is characterized by a dense lymphoid infiltrate composed of lymphoid cells, positive for CD20 and Bcl-2. Imaging can be completely normal. Pathologic findings include a slightly thickened gallbladder wall, with or without gallstones, cholesterolosis and retroperitoneal lymph nodes [76].

## Ultrasound and contrast-enhanced ultrasound

**Fig. 1:**
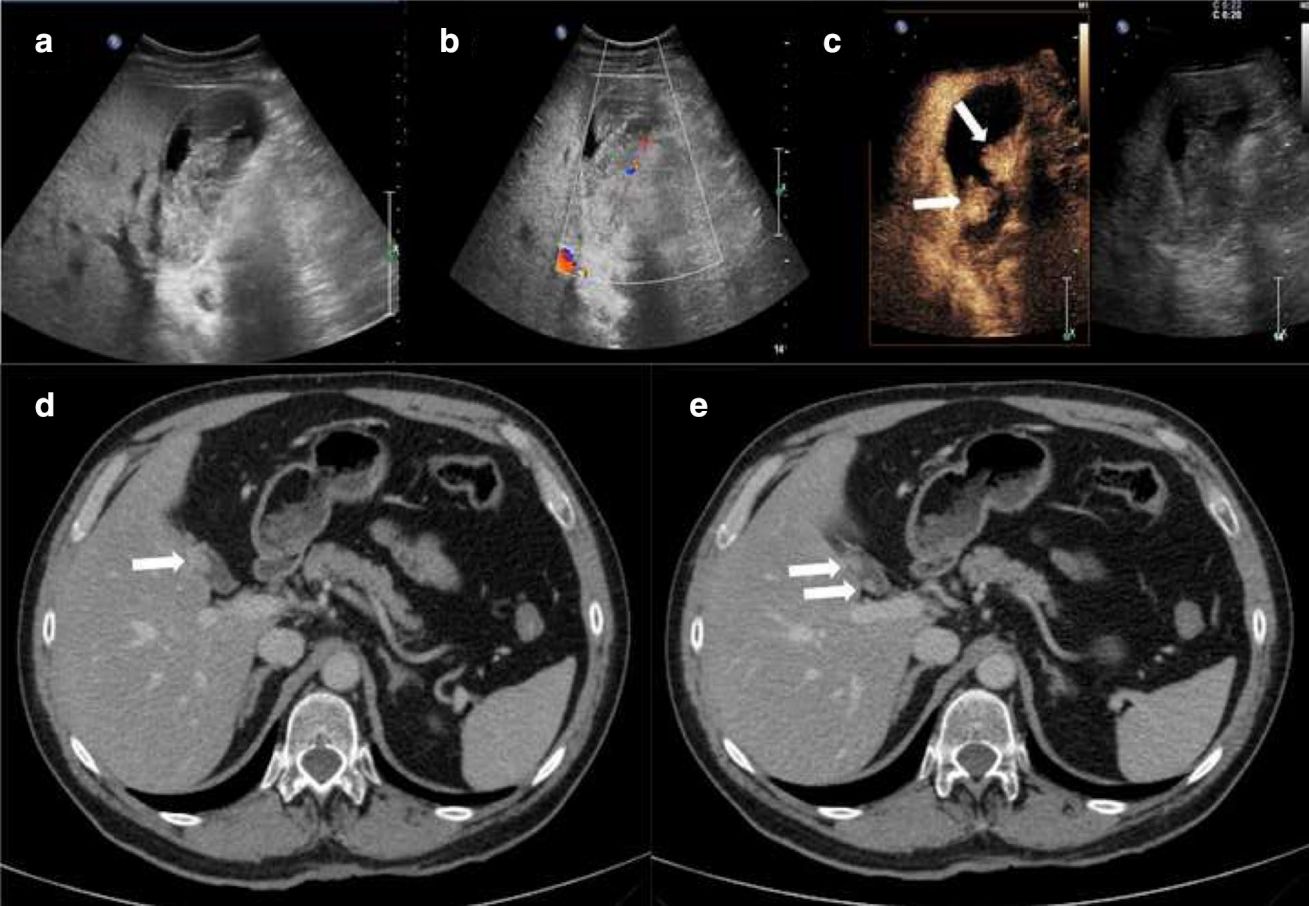
A 56-year-old male hospitalized for right upper abdominal pain with nausea and vomiting. The pain was irradiated to the right scapular region. His medical history included a total excision of dorsal melanoma 2 years before, with no evidence of metastatic disease at the primary staging. The B-mode US (**a**) showed the gallbladder lumen completely filled with heterogeneous content which was difficult to differentiate between tumefactive biliary sludge and parietal mass. The corresponding color-doppler (**b**) revealed no vascular signal within the endoluminal content. CEUS (**c**) demonstrated the vascularization of 2 polypoid lesions (arrows) protruding into the gallbladder lumen with intense contrast enhancement in the arterial phase (20 s). The portal venous phase CT images (**d**, **e**) confirmed two enhancing mural nodules (arrows) of galbladder wall that were histologically proved to be metastases from melanoma

On US, GM appears as a single or multiple mural nodules protruding into the lumen. The nodules usually have a large base, and their echogenicity is generally lower than usually seen in cholesterol and hyperplastic polyps [85]. Usually, a combined finding of biliary sludge and stones can be seen, and B-mode imaging can show the characteristic images of cholecystitis, sometimes due to obstruction of the cystic duct by the tumor mass [85, 86]. A slight mural thickening can be present in combination with luminal vegetations. Phillips et al. [87] separated the US features into four patterns: (A) focal thickening; (B) intraluminal mass without acoustic shadowing; (C) a polypoid or irregular mass; and (D) a gallbladder with indistinct walls. The B-mode US image may show a lumen completely filled with content in which it is difficult to differentiate between tumefactive biliary sludge and parietal masses (Fig. 1a). Color-flow Doppler analysis is non-specific and in fact can show an avascular signal (Fig. 1b), a single central vascular pedicle or multiple spot-like or band-like flow signals. Spectral analysis can reveal an arterial, relatively low-resistance flow, which is a non-specific finding. Differential diagnosis with B-mode and color-flow Doppler analysis between polypoid lesions and biliary sludge is not easy due to these non-specific signs found in the detection of GM [85, 88]. CEUS is able to differentiate between a perfused gallbladder lesion and motionless biliary sludge [85].

The GM US and CEUS pattern is not typical and we can find a variability of enhancement due to the structure of the lesions. For example, metastasis with a large necrotic component may not have enhancement. Although CEUS is nonspecific for the diagnosis of GM, it is useful in distinguishing between solid wall lesions and tumefactive biliary sludge. In these cases, CEUS shows the enhancement absence of the tumefactive biliary sludge in all phases, with a sensitivity and specificity of 100% [85, 88]. CEUS may show hyper-enhanced mural tumor nodules and often the loss of gallbladder wall integrity with infiltration of surrounding liver tissue (Fig. 1C). In some cases, a thin, branching pedicle is recognizable at the center of the enhanced lesion using real-time CEUS; this enhancing pedicle can be perceptible in the first seconds of microbubble arrival at the lesion, or even later [79]. Indeed, with the use of only CEUS it is not possible to make a differential diagnosis between gallbladder adenocarcinoma and GM; it is important to distinguish gallbladder adenocarcinoma from GM by taking an accurate anamnesis of the patient and using other tests that may be helpful. The CEUS features of wash-out within 35 s after administration of the contrast agent, the destruction of gallbladder wall integrity and infiltration of the adjacent liver tissue beneath a solid lesion are highly suggestive features of malignancy [88].

## Computed tomography

Few studies have focused on the CT appearance of GM [89]. Most were case reports and single-center experiences, with an interesting variability in the geographic distribution of the primary tumor [79, 89]. For example, a retrospective analysis by Choi et al. [89] on the Korean prevalence of GM revealed a high incidence of gastrointestinal tract tumors, mostly gastric cancer, followed by HCC and colorectal cancer. On the other hand, studies of Western countries described a higher incidence of melanoma, lung cancer, RCC, breast cancer and non-Hodgkin lymphoma [79, 90]. Compared to US, CT has superior sensitivity and specificity to estimate the extent of the primary disease by identifying lymphadenopathy, peritoneal carcinomatosis and distant organ metastasis, including GM [91]. The CT protocol should include the administration of an intravenous contrast agent. CT assessment of the gallbladder is based on the location of the GM (fundus, body, neck, cystic duct, diffuse), its morphology (infiltrative, polypoid, mass-forming), degree and pattern of enhancement, depth of invasion and signs of concomitant cholecystitis [89]. GM is usually found in the body of the organ, more often with infiltrative morphology [89]. The enhancement pattern can be persistent or show an early wash-in with wash-out as well. The depth of invasion is usually extended to the muscle layer (mT1), the perimuscular connective tissue (mT2) or the serosa (mT3). The mT4 stage, where the lesions spread to two or more organs, is less frequently observed [79]. CT imaging of GM usually reflects the primary tumor behavior [79, 89]. For example, wall thickening with delayed enhancement is more likely associated with adenocarcinomas whereas hypervascular tumors such as melanoma, HCC and RCC usually show early wash-in and wash-out [91]. Of note, Choi et al. identified a specific pattern of growth based on the primary tumor type [89]. In more detail, the infiltrative type of GM was typically observed in adenocarcinoma whereas the polypoid type was mainly associated with non-adenocarcinomatous histology such as melanoma, HCC and RCC [89]. GM has features similar to those of primary gallbladder cancers, especially at an early stage. In fact, infiltrative wall thickening, and polypoid lesions are two of the three main growth patterns of gallbladder cancer [92]. Moreover, features of other gallbladder diseases, including cholecystitis, polyps and adenomyomatosis, may overlap with the features of GM on CT imaging [93] (Fig. 1d, e).

## Magnetic resonance imaging

**Fig. 2:**
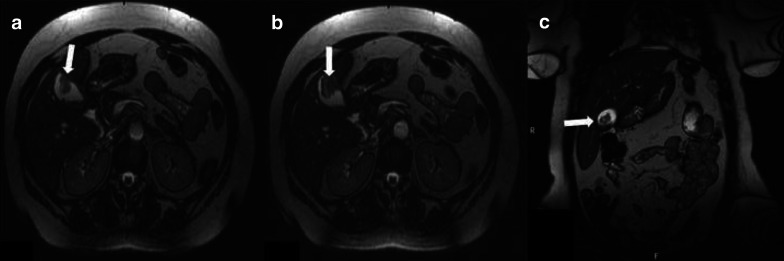
Axial (**a**, **b**) and coronal (**c**) T2-weighted MRI images of an asymptomatic 59-year-old female underwent surgery for a right scapular melanoma 4 years before, with no evidence of metastatic disease at the primary staging. MRI was recommended after a US examination reporting a nonspecific gallbladder mass. The patient was in good general health, nutritional and hydration status. No palpable masses were observed at the clinical examination. MRI images showed a vegetant endoluminal hypointense mass with broad mural base and focal wall thickening. A cholecystectomy was performed and a metastasis from melanoma was histologically confirmed

Few studies are currently available on the role of MRI in GM [79, 94]. In general, the MRI protocol for gallbladder investigation should include thin slice (< 5 mm) axial T1w images, coronal and axial T2w images, 3D-cholangiopancreatic images, axial dynamic contrast enhancement (DCE) images after intravenous gadolinium contrast agent injection and DWI [79, 94]. GM usually shows morphologic features and post-contrast enhancement patterns similar to those of primary gallbladder carcinomas and, for this reason, differential diagnosis is challenging [79]. In fact, GM can appear as single or multiple exophytic masses or polyps arising from the gallbladder wall or infiltrative lesions invading the mucosal, muscular or serosal layer of the gallbladder. Similar to gallbladder adenocarcinomas, GM generally appears iso-hypointense in T1w images and slightly hyperintense in T2w images (Fig. 2) [79, 95]. MRI plays a significant role in the differential diagnosis of GM from malignant melanoma. In fact, melanoma shows high signal intensity on T1w images, due to the low T1 relaxation time of melanin, and appears hypointense on T2w images [95]. MRI-DCE imaging is not specific. GM usually shows inhomogeneous peripheral enhancement in the arterial phase and rapid wash-out in the portal venous phase. DWI evaluates the free Brownian motion of water molecules in tissues and organs. Several studies showed promising results for differentiating benign gallbladder wall thickening-associated conditions from malignancy [52]. For example, a markedly hypointense ADC map signal directly correlates with malignant gallbladder wall pathology, whereas a hyperintense ADC map signal is typically observed with benign conditions. Unfortunately, DWI is not a specific tool for histopathologic image correlation or for detecting the nature of metastasis [52].

## Conclusion

The diagnosis of GM is usually late and rarely isolated. Imaging findings are often associated with other organs involvement in advanced stage of malignancy. For this reason, the, knowledge of the clinical features and different imaging techniques through careful evaluation of the gallbladder can help to achieve early diagnosis and avoid misdiagnosis or false negative results.

## Data Availability

All the original images are available from the corresponding author on reasonable request.

## Abbreviations

ADC: Apparent diffusion coefficient
CECT: Contrast-enhanced CT
CEUS: Contrast-enhanced ultrasound
CT: Computed tomography
DCE: Dynamic contrast enhancement
DWI: Diffusion weighted imaging
GM: Gallbladder metastasis
HCC: Hepatocellular carcinoma
MRI: Magnetic resonance imaging
RCC: Renal cell carcinoma
T1w: T1-weighted
T2w: T2-weighted
US: Ultrasound

## References

[1] Patel S, Zebian B, Gurjar S (2009). An unusual gall-bladder polyp—site of metastatic renal cell carcinoma: a case report. Cases J.

[2] Kawahara T, Ohshiro H, Sekiguchi Z (2010). Gallbladder metastasis from renal cell carcinoma. Case Rep Oncol.

[3] Marone U, Caracò C, Losito S (2007). Laparoscopic cholecystectomy for melanoma metastatic to the gallbladder: is it an adequate surgical procedure? Report of a case and review of the literature. World J Surg Oncol.

[4] Ettahri H, Elomrani F, Elkabous M (2015). Duodenal and gallbladder metastasis of regressive melanoma: a case report and review of the literature. J Gastrointest Oncol.

[5] Das Gupta T, Brasfield R (1964). Metastatic melanoma: a clinicopathological study. Cancer.

[6] Goldin EG (1990). Malignant melanoma metastatic to the gallbladder: case report and review of the literature. Am Surg.

[7] Bowdler DA, Leach RD (1982). Metastatic intrabiliary melanoma. Clin Oncol.

[8] Verbanck JJ, Rutgeerts LJ, van Aelst FJ (1986). Primary malignant melanoma of the gallbladder, metastatic to the common bile duct. Gastroenterology.

[9] Ostick DG, Haqqani MT (1976). Obstructive cholecystitis due to metastatic melanoma. Postgrad Med J.

[10] Langley RGB, Bailey EM, Sober AJ (1997). Acute cholecystitis from metastatic melanoma to the gallbladder in a patient with a low-risk melanoma. Br J Dermatol.

[11] Henriques CQ (1955). A case of secondary melanoma of the gallbladder presenting as acute cholecystitis. Br J Surg.

[12] McFadden PM, Krementz ET, McKinnon WMP, Pararo LL, Ryan RF (1979). Metastatic melanoma of the gallbladder. Cancer.

[13] Dong XD, DeMatos P, Prieto VG, Seigler HF (1999). Melanoma of the gallbladder: a review of cacses seen at Duke University Medical Center. Cancer.

[14] Avila NA, Shawker TH, Fraker D (1994). Color-flow doppler ultrasonography in metastatic melanoma of the gallbladder. J Clin Ultrasound.

[15] Pautler EE, Gallavan EM (1951). Melanoma of brain and gallbladder. Arch Pathol.

[16] Thayer KH, Williams OO, Rowe D (1955). Malignant melanoma of the gallbladder: report of a case and review of the literature. Ariz Med.

[17] Walsh TS (1956). Primary melanoma of the gallbladder with cervical metastasis and fourteen and a half year survival. Cancer.

[18] Jones CH (1961). Malignant melanoma of the gallbladder. J Pathol Bacteriol.

[19] Raffensperger EC, Brason FW, Triano G (1963). Primary melanoma of the gallbladder. Am J Dig Dis.

[20] Peison B, Rabin L (1976). Malignant melanoma of the gallbladder: report of three cases and review of the literature. Cancer.

[21] Sierra-Callejas JL, Warecka K (1976). Primary malignant melanoma of the gallbladder. Virchows Arch A Pathol Anat Histol.

[22] Hatae Y, Kikuchi M, Segawa M (1978). Malignant melanoma of the gallbladder. Pathol Res Pract.

[23] Carle G, Lessells AM, Best PV (1981). Malignant melanoma of the gallbladder: a case report. Cancer.

[24] Anderson JB, Hughes RG, Williamson RN (1983). Malignant melanoma of the gallbladder. Postgrad Med J.

[25] Naguib SE, Aterman K (1984). Presumed primary malignant melanoma of the gallbladder: report of a case and a review of literature. Am J Dermatopathol.

[26] Borja SR, Meyer WR, Cahill JP (1984). Malignant melanoma of the gallbladder: report of a case. Cancer.

[27] Heath DI, Womack C (1988). Primary malignant melanoma of the gallbladder. J Clin Pathol.

[28] Hatanaka N, Miyata M, Kamiike W (1993). Radical resection of primary malignant melanoma of the gallbladder with multiple metastases: report of a case. Surg Today.

[29] Velez AF, Penetrante RB, Spellman JE, Orozco A, Karakousis CP (1995). Malignant melanoma of the gallbladder: report of a case and review of the literature. Am Surg.

[30] Larmi TKI (1960). Malignant melanoma of the gallbladder: report of a case resulting in an external biliary fistula. Acta Chir Scand.

[31] Balthazar EJ, Javors B (1975). Malignant melanoma of the gallbladder. Am J Gastroenterol.

[32] Bundy AL, Ritchie WGM (1982). Ultrasonic diagnosis of metastatic melanoma of the gallbladder presenting as acute cholecystitis. J Clin Ultrasound.

[33] Zemlyn S (1966). Metastatic melanoma of the gallbladder. Radiology.

[34] Herrington JL (1965). Metastatic malignant melanoma of the gallbladder masquerading as cholelithiasis. Am J Surg.

[35] Kazmann HA, Zukaukas CL (1956). Malignant melanoma of the gallbladder. Am J Surg.

[36] Daunt N, King DM (1982). Metastatic melanoma in the biliary tree. Br J Radiol.

[37] Abdelli N, Thiefin G, Diebold MD, Rodriguez JD, Varini E (1996). Endoscopic retrograde cholangiography in a metastatic melanoma of the gallbladder presenting as a gallstone migration. Endoscopy.

[38] Cunningham JJ (1977). Atypical cholesonograms in primary and secondary malignant disease of the biliary tract. J Clin Ultrasound.

[39] Shimkin PM, Soloway MS, Jaffe E (1972). Metastatic melanoma of the gallbladder. Am J Roentgenol Radium Ther Nucl Med.

[40] Hahn ST, Park SH, Choi HS (1993). Ultrasonographic features of metastatic melanoma of the gallbladder. J Clin Ultrasound.

[41] Stutte H, Müllerd PH, d'Hoedt B, Stroebel W (1989). Ultrasonographic diagnosis of melanoma metastases in liver, gallbladder, and spleen. J Ultrasound Med.

[42] Murphy MN, Lorimer SM, Glennon PE (1987). Metastatic melanoma of the gallbladder: a case report and review of the literature. J Surg Oncol.

[43] Katz SC, Bowne WB, Wolchok JD (2007). Surgical management of melanoma of the gallbladder: a report of 13 cases and review of the literature. Am J Surg.

[44] Samplaski MK, Rosato EL, Witkiewicz AK, Mastrangelo MJ, Berger AC (2008). Malignant melanoma of the gallbladder: a report of two cases and review of the literature. J Gastrointest Surg.

[45] Giannini I, Cutrignelli DA, Resta L, Gentile A, Vincenti L (2016). Metastatic melanoma of the gallbladder: report of two cases and a review of the literature. Clin Exp Med.

[46] Ercolino GR, Guglielmi G, Pazienza L, Urbano F, Palladino D, Simeone A (2018). Gallbladder and small bowel metastasis of regressive melanoma: a case report. BJR Case Rep.

[47] Nojima H, Cho A, Yamamoto H (2008). Renal cell carcinoma with unusual metastasis to the gallbladder. J Hepatobiliary Pancreat Surg.

[48] Kitamura H, Kurokawa M, Inaki N (2018). Gallbladder metastasis from renal cell carcinoma. Indian J Surg.

[49] Castro Ruiz C, Pedrazzoli C, Bonacini S (2016). Gallbladder’s clear cell renal carcinoma metastasis: a case report. Int J Surg Case Rep.

[50] Kinoshita O, Dohi M, Horii Y, Ikai A, Kitamori T, Yamashita T (2019). Simultaneous resection of gastric and gallbladder metastasis from renal cell carcinoma treated by laparoscopic and endoscopic cooperative surgery: a case report. Surg Case Rep.

[51] Costa Neves M, Neofytou K, Giakoustidis A (2016). Two cases of gallbladder metastasis from renal cell carcinoma and review of literature. World J Surg Oncol.

[52] Ueda I, Aoki T, Oki H (2015). Gallbladder metastasis from renal cell carcinoma: a case report with review of the literature. Magn Reson Med Sci.

[53] Jeong YS, Han HS, Lim SN (2012). Gallbladder metastasis of non-small cell lung cancer presenting as acute cholecystitis. Chin J Cancer Res.

[54] Gutknecht DR (1998). Metastatic lung cancer presenting as cholecystitis. Am J Gastroenterol.

[55] Nassenstein K, Kissler M (2004). Gallbladder metastasis of non-small cell lung cancer. Onkologie.

[56] Jeong HT, Yun M, Hong HS, Lee JD, Kim KW (2010). Unusual gallbladder metastasis from non-small-cell lung cancer detected by F-18 FDG PET/CT with intravenous contrast enhancement. Clin Nucl Med.

[57] Bezpalko K, Mohamed MA, Mercer L, McCann M, Elghawy K, Wilson K (2015). Concomitant endometrial and gallbladder metastasis in advanced multiple metastatic invasive lobular carcinoma of the breast: a rare case report. Int J Surg Case Rep.

[58] Nobori C, Kodai S, Kanazawa A (2019). A case of gallbladder metastasis from breast cancer with acute calculous cholecystitis. Gan To Kagaku Ryoho.

[59] Zagouri F, Sergentanis TN, Koulocheri D (2007). Bilateral synchronous breast carcinomas followed by a metastasis to the gallbladder: a case report. World J Surg Oncol.

[60] Essola B, Malvaux P, Landenne J (2012). Gallbladder metastasis from breast carcinoma: a new case report. Rev Med Brux.

[61] Belhachmi A, Ouazni M, Rajae Y (2014). Acute cholecystitis as a rare presentation of metastatic breast carcinoma of the gallbladder: a case report and review of the literature. Pan Afr Med J.

[62] Mouchli M, Grider DJ, Yeaton P (2019). Gallbladder metastases: a report of two cases. Case Rep Oncol.

[63] Molina-Barea R, Rios-Peregrina RM, Slim M, Calandre EP, Hernández-García MD, Jimenez-Rios JA (2014). Lobular breast cancer metastasis to the colon, the appendix and the gallbladder. Breast Care (Basel).

[64] Doval DC, Komal B, Keechelat P, Sharma JB, Vaid AK, Hazarika D (2006). Breast carcinoma with metastasis to the gallbladder: an unusual case report with a short review of literature. Hepatobiliary Pancreat Dis Int.

[65] Murguia E, Quiroga D, Canteros G, SanMartino C, Barreiro M, Herrera J (2006). Gallbladder metastases from ductal papillary carcinoma of the breast. J Hepatobiliary Pancreat Surg.

[66] Boari B, Pansini G, Pedriali M, Cavazzini L, Manfredini R (2005). Acute cholecystitis as a presentation of metastatic breast carcinoma of the gallbladder: a case report. J Am Geriatr Soc.

[67] Manouras A, Lagoudianakis EE, Genetzakis M, Pararas N, Papadima A, Kekis PB (2008). Metastatic breast carcinoma initially presenting as acute cholecystitis: a case report and review of the literature. Eur J Gynaecol Oncol.

[68] Markelov A, Taheri H, Vunnamadala K, Ibrahim G (2011). Biliary dyskinesia as a rare presentation of metastatic breast carcinoma of the gallbladder: a case report. Case Rep Pathol.

[69] Wakasugi M, Ueshima S, Akamatsu H (2012). Gallbladder metastasis from hepatocellular carcinoma: report of a case and review of literature. Int J Surg Case Rep.

[70] Lane JE, Walker AN (2002). Metastatic hepatocellular carcinoma of the gallbladder. Dig Surg.

[71] Ando K, Sakamoto Y (2009). A case of gallbladder metastasis from hepatocellular carcinoma. Jpn J Clin Oncol.

[72] Murakami M, Kobayashi S, Marubashi S (2010). Isolated metastasis to the gallbladder from hepatocellular carcinoma. Hepatol Res.

[73] Kanzaki R, Yamada T, Gotoh K (2011). Surgical resection for hepatocellular carcinoma with metastasis to the gallbladder: report of a case. Surg Today.

[74] Ooe Y, Tsukada T, Yamasaki Y, Kaji M, Shimizu K (2019). A case of synchronous and solitary gallbladder metastasis from gastric cancer. Gan To Kagaku Ryoho.

[75] Bılıcı A, Şeker M, Ustaalıoğlu BBO (2012). Gallbladder metastasis secondary to gastric cancer as a first site of recurrence presented with acute cholecystitis: case report and literature review. Turk J Gastroenterol.

[76] Muszynska C, Lundgren L, Andersson R (2019). Incidental metastases and lymphoma of the gallbladder—an analysis of ten rare cases identified from a large national database. Scand J Gastroenterol.

[77] Patnana M, Bronstein Y, Szklaruk J (2011). Multimethod imaging, staging, and spectrum of manifestations of metastatic melanoma. Clin Radiol.

[78] Gupta P, Marodia Y, Bansal A (2020). Imaging-based algorithmic approach to gallbladder wall thickening. World J Gastroenterol.

[79] Barretta ML, Catalano O, Setola SV, Granata V, Marone U, Gallipoli AD (2011). Gallbladder metastasis: spectrum of imaging findings. Abdom Imaging.

[80] Xie XH, Xu HX, Xie XY (2010). Differential diagnosis between benign and malignant gallbladder diseases with real-time contrast-enhanced ultrasound. Eur Radiol.

[81] Takagi K, Kawase K, Minoshima K (2019). Gallbladder metastasis from renal cell carcinoma: a case report and literature review. Urol Case Rep.

[82] O'Shaughnessy J (2005). Extending survival with chemotherapy in metastatic breast cancer. Oncologist.

[83] Nakashima T, Okuda K, Kojiro M (1983). Pathology of hepatocellular carcinoma in Japan. 232 Consecutive cases autopsied in ten years. Cancer.

[84] Yoon WJ, Yoon YB, Kim YJ, Ryu JK, Kim YT (2009). Metastasis to the gallbladder: a single-center experience of 20 cases in South Korea. World J Gastroenterol.

[85] Cocco G, Basilico R, Delli Pizzi A (2021). Gallbladder polyps ultrasound: what the sonographer needs to know. J Ultrasound.

[86] Tana M, Tana C, Cocco G, Iannetti G, Romano M, Schiavone C (2015). Acute acalculous cholecystitis and cardiovascular disease: a land of confusion. J Ultrasound.

[87] Phillips G, Pochaczevsky R, Kumari S (1982). Ultrasound patterns of metastatic tumors in the gallbladder. J Clin Ultrasound.

[88] Sidhu PS, Vito Cantisani V, Christoph F (2017). The EFSUMB guidelines and recommendations for the clinical practice of contrast-enhanced ultrasound (CEUS) in non-hepatic applications: update. Ultraschall in Med.

[89] Choi WS, Kim SH, Lee ES (2014). CT findings of gallbladder metastases: emphasis on differences according to primary tumors. Korean J Radiol.

[90] Rehani B, Strohmeyer P, Jacobs M (2006). Gallbladder metastasis from malignant melanoma: diagnosis with FDG PET/CT. Clin Nucl Med.

[91] Horton KM, Fishman EK (2003). Current role of CT in imaging of the stomach. Radiographics.

[92] Ishiguro S, Onaya H, Esaki M (2012). Mucin-producing carcinoma of the gallbladder: evaluation by magnetic resonance cholangiopancreatography in three cases. Korean J Radiol.

[93] Hickman L, Contreras C (2019). Gallbladder cancer: diagnosis, surgical management, and adjuvant therapies. Surg Clin N Am.

[94] Catalano OA, Sahani DV, Kalva SP (2008). MR imaging of the gallbladder: a pictorial essay. Radiographics.

[95] Chatterjee A, Lopes Vendrami C, Nikolaidis P (2019). Uncommon intraluminal tumors of the gallbladder and biliary tract: spectrum of imaging appearances. Radiographics.

